# Cortical circuits for perceptual inference

**DOI:** 10.1016/j.neunet.2009.07.023

**Published:** 2009-10

**Authors:** Karl Friston, Stefan Kiebel

**Affiliations:** The Wellcome Trust Centre of Neuroimaging, University College London, Queen Square, London WC1N 3BG, United Kingdom

**Keywords:** Generative models, Predictive coding, Hierarchical, Dynamic, Nonlinear, Circuits, Variational, Birdsong, Free-energy

## Abstract

This paper assumes that cortical circuits have evolved to enable inference about the causes of sensory input received by the brain. This provides a principled specification of *what* neural circuits have to achieve. Here, we attempt to address *how* the brain makes inferences by casting inference as an optimisation problem. We look at how the ensuing recognition dynamics could be supported by directed connections and message-passing among neuronal populations, given our knowledge of intrinsic and extrinsic neuronal connections. We assume that the brain models the world as a dynamic system, which imposes causal structure on the sensorium. Perception is equated with the optimisation or inversion of this internal model, to explain sensory input. Given a model of how sensory data are generated, we use a generic variational approach to model inversion to furnish equations that prescribe recognition; i.e., the dynamics of neuronal activity that represents the causes of sensory input. Here, we focus on a model whose hierarchical and dynamical structure enables simulated brains to recognise and predict sequences of sensory states. We first review these models and their inversion under a variational free-energy formulation. We then show that the brain has the necessary infrastructure to implement this inversion and present stimulations using synthetic birds that generate and recognise birdsongs.

## 1 Introduction

This paper looks at the functional architecture of cortical circuits from the point of view of perception; namely, the fitting or inversion of internal models of sensory data by the brain. Critically, the nature of this inversion lends itself to a relatively simple neural network implementation that shares many formal similarities with real cortical hierarchies in the brain. The basic idea that the brain uses hierarchical inference has been described in a series of papers (Friston, 2005; Friston, Kilner, & Harrison, 2006; Mumford, 1992; Rao & Ballard, 1998). These papers suggest that the brain uses *empirical* Bayes for inference about its sensory input, given the hierarchical organisation of cortical systems. Here, we focus on how neural networks could be configured to invert these models and deconvolve sensory causes from sensory input.

This paper comprises three sections. In the first, we introduce a free-energy formulation of model inversion or perception, which is then applied to a specific class of models that we assume the brain uses — hierarchical dynamic models. An important aspect of these models is their formulation in generalised coordinates of motion. This lends them a hierarchical form in both structure and dynamics, which can be exploited during inversion. In the second section, we show how inversion can be formulated as a simple gradient descent using neuronal networks and relate these to cortical circuits in the brain. In the final section, we consider how evoked brain responses might be understood in terms of perceptual inference and categorisation, using the schemes of the preceding section.

## 2 The free-energy formulation

This section considers the problem of inverting generative models of sensory data and provides a summary of the material in Friston (2008). This problem is addressed using *ensemble learning* or *Variational Bayes*. These are generic approaches to model inversion that provide an approximation to the conditional density $p\left(\vartheta |\overset{˜}{y}\right)$ on some causes ϑ of generalised sensory input, $\overset{˜}{y}={\left[y,y^{\prime },y^{\prime \prime },\ldots \right]}^{T}$. Generalised input (e.g., the intensity of photoreceptor stimulation) includes the input, its velocity, acceleration, jerk, *etc*. Causes are quantities in the environment that generate sensory input (e.g., the orientation of an object in the visual field). The approximation of the conditional density (i.e., the probability of a particular set of causes given sensory input) is achieved by optimising a recognition density q(ϑ) with respect to a bound on the surprise or negative log-evidence $-\ln p\left(\overset{˜}{y}\right)$ of the sensory input, as we will see next (Feynman, 1972; Friston, 2005; Friston et al., 2006; Hinton & von Cramp, 1993; MacKay, 1995; Neal & Hinton, 1998). This bound is called free-energy

(1) $$F=C-\ln p\left(\overset{˜}{y}\right)$$

$$C={\langle \ln\frac{q\left(\vartheta \right)}{p\left(\vartheta |\overset{˜}{y}\right)}\rangle }_{q}\text{.}$$

The free-energy comprises a cross-entropy or divergence term C≥0 and surprise. By Gibb’s inequality, the divergence is greater than zero, with equality when $q\left(\vartheta \right)=p\left(\vartheta |\overset{˜}{y}\right)$; i.e., when the recognition density is the posterior or conditional density on the causes of sensory input. The recognition density can be optimised to minimise this bound and implicitly minimise the divergence between the recognition density and the conditional density we seek (Friston, 2005; Hinton & von Cramp, 1993; MacKay, 1995; Neal & Hinton, 1998). In summary, the recognition density, induces a free-energy bound, which converts a difficult integration problem (inherent in computing the exact conditional density) into an easier optimisation problem.

The bound can be evaluated easily because it is a function of q(ϑ) and some generative model $p\left(\overset{˜}{y},\vartheta \right)$ entailed by the brain

(2) $$F={\langle \ln q\left(\vartheta \right)-\ln p\left(\vartheta |\overset{˜}{y}\right)-\ln p\left(\overset{˜}{y}\right)\rangle }_{q}={\langle \ln q\left(\vartheta \right)\rangle }_{q}+{\langle U\left(\vartheta \right)\rangle }_{q}$$

$$U\left(\vartheta \right):=-\ln p\left(\overset{˜}{y},\vartheta \right)\text{.}$$

Here, we have expressed the free-energy in terms of the negentropy of q(ϑ) and an expected Gibb’s energy — U(ϑ). This energy is usually specified in terms of a likelihood and prior; $U\left(\vartheta \right)=-\ln p\left(\overset{˜}{y}|\vartheta \right)-\ln p\left(\vartheta \right)$, which define a generative model. This is important because it shows that we need a generative model in order to evaluate free-energy. The likelihood model just quantifies the probability of any sensations, given their cause; while the prior model encodes prior beliefs about the probability of those causes being present. It is fairly easy to show that minimising free-energy corresponds to finding a recognition density that predicts sensory input accurately, while suppressing its complexity.

If we assume that the recognition density $q\left(\vartheta \right)=N\left(\overset{˜}{\mu },\overset{˜}{\Sigma }\right)$ is Gaussian (the Laplace assumption), then we can express free-energy in terms of its sufficient statistics (i.e., its mean and covariance: $\overset{˜}{\mu },\overset{˜}{\Sigma }$)

(3) $$F=U\left(\overset{˜}{\mu }\right)-\frac{1}{2}tr\left(\overset{˜}{\Sigma }\nabla^{2}U\right)-\frac{1}{2}\ln\left|\overset{˜}{\Sigma }\right|-\frac{n}{2}\ln2\pi e\text{.}$$

Here n is the number of unknown causes. We can now minimise free-energy w.r.t. the conditional covariances by finding the value that renders its gradient zero

(4) $$F_{\Sigma }=-\frac{1}{2}\overset{˜}{\Pi }-\frac{1}{2}\nabla^{2}U=0\Rightarrow$$

$$\overset{˜}{\Pi }=\nabla^{2}U\left(\overset{˜}{\mu }\right)$$

where a subscript means differentiation; i.e., $F_{\Sigma }=\partial F/\partial \Sigma$ is the free-energy gradient w.r.t. the conditional covariance, Here, the conditional precision $\overset{˜}{\Pi }={\overset{˜}{\Sigma }}^{-1}$ is the inverse covariance. Critically, the conditional precision is just a function of the mean and does not have to be encoded explicitly. This means we can simplify the expression for free-energy by eliminating the curvatures $\nabla^{2}U$ of Gibb’s energy

(5) $$F=U\left(\overset{˜}{\mu }\right)-\frac{1}{2}\ln\left|\overset{˜}{\Sigma }\right|-\frac{n}{2}\ln2\pi \text{.}$$

Now, the only unknown quantities are the conditional means of the causes, which only have to minimise Gibb’s energy because this is the only term that depends on them. In this paper, we will focus on time-varying causes or states of the environment: $\overset{˜}{u}\left(t\right)\subset \vartheta$. The values we seek are the solutions to the following differential equations.

(6) $$\begin{array}{ccc} {\overset{˙}{\overset{˜}{\mu }}}^{u} & = & D{\overset{˜}{\mu }}^{u}-U_{\overset{˜}{u}}\\ & \Leftrightarrow & \\ {\overset{˙}{\mu }}^{u} & = & {\mu {}^{\prime }}^{u}-U_{u}\\ {\overset{˙}{\mu }{}^{\prime }}^{u} & = & {\mu {}^{\prime \prime }}^{u}-U_{u}^{\prime }\\ {\overset{˙}{\mu }{}^{\prime \prime }}^{u} & = & {\mu {}^{\prime \prime \prime }}^{u}-U_{u}^{\prime \prime }\\ \vdots \text{.} & & \end{array}$$

This solution (which is stationary in a frame of reference that moves with its generalised motion), minimises free-energy

(7) $${\overset{˙}{\overset{˜}{\mu }}}^{u}-D{\overset{˜}{\mu }}^{u}=0\Rightarrow$$

$$U_{\overset{˜}{u}}=0\Rightarrow F_{\overset{˜}{u}}=0\text{.}$$

This construction ensures that when Gibb’s energy is minimised and $U_{\overset{˜}{u}}=0$, the mean of the motion is the motion of the mean; i.e., ${\overset{˙}{\overset{˜}{\mu }}}^{u}=D{\overset{˜}{\mu }}^{u}$. Here D is a derivative matrix operator with identity matrices along the first leading diagonal.

Eq. (7) prescribes recognition dynamics that track time-varying causes or states of the world and can be thought of as a gradient descent in a moving frame of reference. The recognition dynamics for time-invariant causes (i.e., parameters θ⊂ϑ, like rate constants) have a different form, because we know *a priori* their generalised motion is zero. In this paper, we will assume the parameters have already been learnt and focus on recognising hidden states of the environment. In summary, we have derived recognition dynamics for expected environmental states, which cause sensations. The solution to these equations minimise Gibb’s energy and (under the Laplace assumption) free-energy, which is an upper bound on their surprise. Finding these solutions corresponds to perceptual inference. The precise form of Eq. (7) depends on the generative model that defines Gibb’s energy. Next, we examine forms associated with hierarchical dynamic models.

### 2.1 Hierarchical dynamic models

This section introduces a general class of generative models that the brain may use for perception. We will start with simple dynamic models and then deal with hierarchical cases later. Consider a state-space model that describes the evolution of states in the world and how they map to sensory input

(8) y=g(x,v)+z

$$\overset{˙}{x}=f\left(x,v\right)+w\text{.}$$

Here, the functions f and g are parameterised by θ⊂ϑ (which are omitted from the following expressions for clarity). These functions correspond to equations of motion and an observer function, respectively. The states v⊂u are variously referred to as sources or causal states. The hidden states x⊂u mediate the influence of causal states on sensory data and endow the system with memory. We assume the random fluctuations z are analytic, such that the covariance of $\overset{˜}{z}={\left[z,z^{\prime },z^{\prime \prime },\ldots \right]}^{T}$ is well defined; similarly for state noise, w(t), which represents random fluctuations on the motion of the hidden states. Under local linearity assumptions, the generalised motion of the data or response $\overset{˜}{y}={\left[y,y^{\prime },y^{\prime \prime },\ldots \right]}^{T}$ is given by

(9) $$\begin{array}{c} \begin{array}{ccc} y & = & g\left(x,v\right)+z\\ y^{\prime } & = & g_{x}x^{\prime }+g_{v}v^{\prime }+z^{\prime }\\ y^{\prime \prime } & = & g_{x}x^{\prime \prime }+g_{v}v^{\prime \prime }+z^{\prime \prime }\\ \vdots & & \end{array}\;\begin{array}{ccc} \overset{˙}{x} & = & x^{\prime }=f\left(x,v\right)+w\\ {\overset{˙}{x}}^{\prime } & = & x^{\prime \prime }=f_{x}x^{\prime }+f_{v}v^{\prime }+w^{\prime }\\ {\overset{˙}{x}}^{\prime \prime } & = & x^{\prime \prime \prime }=f_{x}x^{\prime \prime }+f_{v}v^{\prime \prime }+w^{\prime \prime }\\ \vdots \text{.} & & \end{array} \end{array}$$

We can write this generalised state-space model more compactly as

(10) $$\overset{˜}{y}=\overset{˜}{g}+\overset{˜}{z}$$

$$D\overset{˜}{x}=\overset{˜}{f}+\overset{˜}{w}$$

where the predicted response $\overset{˜}{g}={\left[g,g^{\prime },g^{\prime \prime },\ldots \right]}^{T}$ and motion $\overset{˜}{f}={\left[f,f^{\prime },f^{\prime \prime },\ldots \right]}^{T}$ are

(11) $$\begin{array}{c} \begin{array}{ccc} g & = & g\left(x,v\right)\\ g^{\prime } & = & g_{x}x^{\prime }+g_{v}v^{\prime }\\ g^{\prime \prime } & = & g_{x}x^{\prime \prime }+g_{v}v^{\prime \prime }\\ \vdots & & \end{array}\;\begin{array}{ccc} f & = & f\left(x,v\right)\\ f^{\prime } & = & f_{x}x^{\prime }+f_{v}v^{\prime }\\ f^{\prime \prime } & = & f_{x}x^{\prime \prime }+f_{v}v^{\prime \prime }\\ \vdots \text{.} & & \end{array} \end{array}$$

Gaussian assumptions about the fluctuations $p\left(\overset{˜}{z}\right)=N\left(\overset{˜}{z}:0,{\overset{˜}{\Sigma }}^{z}\right)$ provide the form of the likelihood, $p\left(\overset{˜}{y}|\overset{˜}{x},\overset{˜}{v}\right)$. Similarly, Gaussian assumptions about state noise $p\left(\overset{˜}{w}\right)=N\left(\overset{˜}{w}:0,{\overset{˜}{\Sigma }}^{w}\right)$ specify empirical priors, $p\left(\overset{˜}{x}|\overset{˜}{v}\right)$ in terms of predicted motion

(12) $$\begin{array}{ccc} p\left(\overset{˜}{y},\overset{˜}{x},\overset{˜}{v}\right) & = & p\left(\overset{˜}{y}|\overset{˜}{x},\overset{˜}{v}\right)p\left(\overset{˜}{x},\overset{˜}{v}\right)\\ p\left(\overset{˜}{x},\overset{˜}{v}\right) & = & p\left(\overset{˜}{x}|\overset{˜}{v}\right)p\left(\overset{˜}{v}\right)\\ p\left(\overset{˜}{y}|\overset{˜}{x},\overset{˜}{v}\right) & = & N\left(\overset{˜}{y}:\overset{˜}{g},{\overset{˜}{\Sigma }}^{z}\right)\\ p\left(\overset{˜}{x}|\overset{˜}{v}\right) & = & N\left(D\overset{˜}{x}:\overset{˜}{f},{\overset{˜}{\Sigma }}^{w}\right)\text{.} \end{array}$$

The covariances ${\overset{˜}{\Sigma }}^{z}$ and ${\overset{˜}{\Sigma }}^{w}$ or precisions ${\overset{˜}{\Pi }}^{z}\left(\lambda \right)$ and ${\overset{˜}{\Pi }}^{w}\left(\lambda \right)$ are functions of precision parameters, λ⊂ϑ, which control the amplitude and smoothness of random fluctuations. Generally, these covariances factorise; ${\overset{˜}{\Sigma }}^{\cdot }=\Sigma^{\cdot }⊗R^{\cdot }$ into a covariance proper and a matrix of correlations $R^{\cdot }$ among generalised motion that encodes an autocorrelation function.

#### 2.1.1 Hierarchical forms

Hierarchical dynamic models with m levels have the following form, which generalises the m=1 model above

(13) $$\begin{array}{ccc} y & = & g\left(x^{\left(1\right)},v^{\left(1\right)}\right)+z^{\left(1\right)}\\ {\overset{˙}{x}}^{\left(1\right)} & = & f\left(x^{\left(1\right)},v^{\left(1\right)}\right)+w^{\left(1\right)}\\ \vdots & & \\ v^{\left(i-1\right)} & = & g\left(x^{\left(i\right)},v^{\left(i\right)}\right)+z^{\left(i\right)}\\ {\overset{˙}{x}}^{\left(i\right)} & = & f\left(x^{\left(i\right)},v^{\left(i\right)}\right)+w^{\left(i\right)}\\ \vdots & & \\ v^{\left(m\right)} & = & z^{\left(m+1\right)}\text{.} \end{array}$$

Again, $f^{\left(i\right)}=f\left(x^{\left(i\right)},v^{\left(i\right)}\right)$ and $g^{\left(i\right)}=g\left(x^{\left(i\right)},v^{\left(i\right)}\right)$ are continuous nonlinear functions of the states. The innovations $z^{\left(i\right)}$ and $w^{\left(i\right)}$ are conditionally independent fluctuations that enter each level of the hierarchy. These play the role of observation error or noise at the first level and induce random fluctuations in the states at higher levels. The causal states $v={\left[v^{\left(1\right)},\ldots ,v^{\left(m\right)}\right]}^{T}$ link levels, whereas the hidden states $x={\left[x^{\left(1\right)},\ldots ,x^{\left(m\right)}\right]}^{T}$ link dynamics over time. In hierarchical form, the output of one level acts as an input to the next. Inputs from higher levels can enter nonlinearly into the state equations and can be regarded as changing its control parameters to produce quite complicated generalised convolutions with deep (i.e., hierarchical) structure.

In summary, hierarchical dynamic models are about as complicated as one could imagine; they comprise causal and hidden states, whose dynamics can be coupled with arbitrary (analytic) nonlinear functions. Furthermore, these states can have random fluctuations with unknown amplitude and arbitrary (analytic) autocorrelation functions. A key aspect of these models is their hierarchical form, which induces empirical priors on the causal states. See Kass and Steffey (1989) for a discussion of approximate Bayesian inference in conditionally independent hierarchical models of static data.

#### 2.1.2 Energy functions

We can now write down Gibb’s energy for these generative models, which has a simple quadratic form (ignoring constants)

(14) $$\begin{array}{ccc} U & = & \ln p\left(\overset{˜}{y},\overset{˜}{x},\overset{˜}{v},\theta ,\lambda \right)=\frac{1}{2}\ln\left|\overset{˜}{\Pi }\right|-\frac{1}{2}{\overset{˜}{\varepsilon }}^{T}\overset{˜}{\Pi }\overset{˜}{\varepsilon }\\ \overset{˜}{\Pi } & = & \left[\begin{array}{cc} {\overset{˜}{\Pi }}^{z} & 0\\ 0 & {\overset{˜}{\Pi }}^{w} \end{array}\right]\\ \overset{˜}{\varepsilon } & = & \left[\begin{array}{c} {\overset{˜}{\varepsilon }}^{v}=\overset{˜}{y}-\overset{˜}{g}\\ {\overset{˜}{\varepsilon }}^{x}=D\overset{˜}{x}-\overset{˜}{f} \end{array}\right]\text{.} \end{array}$$

The auxiliary variables $\overset{˜}{\varepsilon }\left(t\right)$ comprise prediction errors for the generalised response and motion of hidden states, where $\overset{˜}{g}\left(t\right)$ and $\overset{˜}{f}\left(t\right)$ are the respective predictions, whose precision is encoded by $\overset{˜}{\Pi }\left(\lambda \right)$. These prediction errors provide a compact way to express Gibb’s energy and, as we will see below, lead to very simple recognition schemes. For hierarchical models, the prediction error on the response is supplemented with prediction errors on the causal states

(15) $$\varepsilon^{v}=\left[\begin{array}{c} y\\ v^{\left(1\right)}\\ \vdots \\ v^{\left(m\right)} \end{array}\right]-\left[\begin{array}{c} g^{\left(1\right)}\\ g^{\left(2\right)}\\ \vdots \\ 0 \end{array}\right]\text{.}$$

Note that the data enter the prediction error at the lowest level. At intermediate levels, the prediction errors, $v^{\left(i-1\right)}-g^{\left(i\right)}$ mediate empirical priors on the causal states.

### 2.2 Summary

In this section, we have seen how the inversion of dynamic models can be formulated as an optimisation of free-energy. By assuming a Gaussian (Laplace) approximation to the conditional density, one can reduce optimisation to finding the conditional means of the unknown causes of sensory data. This can be formulated as a gradient ascent in a frame of reference that moves along the path encoded in generalised coordinates (Eq. (6)). The only thing needed to implement this recognition scheme is Gibb’s energy, which is specified by a generative model. We have looked at hierarchical dynamic models, whose form provides empirical priors or constraints on inference at both a structural and dynamic level (Eq. (14)). The *structural* priors arise from coupling different levels of the hierarchy with causal states and the *dynamic* priors emerge by coupling different levels of generalised motion of the hidden states. We can now look at the recognition dynamics entailed by these models, in the context of neuronal processes in the brain.

## 3 Hierarchical models in the brain

A key architectural principle of the brain is its hierarchical organisation (Felleman & Van Essen, 1991; Maunsell & van Essen, 1983; Zeki & Shipp, 1988).  This has been established most thoroughly in the visual system, where lower (primary) areas receive sensory input and higher areas adopt a multimodal or associational role. The neurobiological notion of a hierarchy rests upon the distinction between forward and backward connections (Angelucci et al., 2002; Felleman & Van Essen, 1991; Murphy & Sillito, 1987; Rockland & Pandya, 1979; Sherman & Guillery, 1998). This distinction is based upon the specificity of cortical layers that are the predominant sources and origins of extrinsic connections. Forward connections arise largely in superficial pyramidal cells, in supra-granular layers and terminate on spiny stellate cells of layer four in higher cortical areas (DeFelipe, Alonso-Nanclares, & Arellano, 2002; Felleman & Van Essen, 1991). Conversely, backward connections arise largely from deep pyramidal cells in infra-granular layers and target cells in the infra- and supra-granular layers of lower cortical areas. Intrinsic connections mediate lateral interactions between neurons that are a few millimetres away. There is a key functional asymmetry between forward and backward connections that renders backward connections more modulatory or nonlinear in their effects on neuronal responses (Sherman & Guillery, 1998; see also Hupe et al., 1998). This is consistent with the deployment of voltage-sensitive NMDA receptors in supra-granular layers that are targeted by backward connections (Rosier, Arckens, Orban, & Vandesande, 1993). Typically, the synaptic dynamics of backward connections have slower time constants. This has led to the notion that forward connections are driving and illicit an obligatory response in higher levels, whereas backward connections have both driving and modulatory effects and operate over larger spatial and temporal scales. This hierarchical aspect of the brain’s functional anatomy speaks to hierarchical models of sensory input. We now consider how this functional architecture can be understood under the inversion of hierarchical models by the brain.

### 3.1 Perceptual inference

If we assume that the activity of neurons encode the conditional mean of external states causing sensory data, then Eq. (6) specifies the neuronal dynamics entailed by recognising states of the world from sensory data. Using Gibb’s energy in Eq. (14) we have

(16) $${\overset{˙}{\overset{˜}{\mu }}}^{u}=D{\overset{˜}{\mu }}^{u}-U_{\overset{˜}{u}}=D{\overset{˜}{\mu }}^{u}-{\overset{˜}{\varepsilon }}_{u}^{T}\xi$$

$$\begin{array}{ccc} \xi & = & \overset{˜}{\Pi }\overset{˜}{\varepsilon }=\overset{˜}{\varepsilon }-\Lambda \xi \\ \overset{˜}{\Pi } & = & \left[\begin{array}{cc} {\overset{˜}{\Pi }}^{z} & \\ & {\overset{˜}{\Pi }}^{w} \end{array}\right]\text{.} \end{array}$$

Eq. (16) describes how neuronal states self-organise, when exposed to sensory input. Its form is quite revealing and suggests two distinct populations of neurons; causal or hidden *state-units* whose activity encodes ${\overset{˜}{\mu }}^{u}:=\overset{˜}{\mu }\left(t\right)$ and *error-units* encoding precision-weighted prediction error $\xi =\overset{˜}{\Pi }\overset{˜}{\varepsilon }$, with one error-unit for each state. Furthermore, the activities of error-units are a function of the states and the dynamics of state-units are a function of prediction error. This means the two populations pass messages to each other and to themselves. The messages passed within the states, $D\overset{˜}{\mu }$ mediate empirical priors on their motion, while −Λξ mediates precision-dependent modulation of prediction errors. The matrix $\Lambda =\overset{˜}{\Sigma }-1$ can be thought of encoding self-inhibition, which is modulated by precision (where precision might be encoded by neuromodulatory neurotransmitters like dopamine and acetylcholine).

### 3.2 Hierarchical message-passing

If we unpack these equations, we can see the hierarchical nature of the implicit message-passing

(17) $$\begin{array}{ccc} {\overset{˙}{\overset{˜}{\mu }}}^{\left(i\right)v} & = & D{\overset{˜}{\mu }}^{\left(i\right)v}-{\overset{˜}{\varepsilon }}_{v}^{\left(i\right)T}\xi^{\left(i\right)}-\xi^{\left(i+1\right)v}\\ {\overset{˙}{\overset{˜}{\mu }}}^{\left(i\right)x} & = & D{\overset{˜}{\mu }}^{\left(i\right)x}-{\overset{˜}{\varepsilon }}_{x}^{\left(i\right)T}\xi^{\left(i\right)}\\ \xi^{\left(i\right)v} & = & {\overset{˜}{\mu }}^{\left(i-1\right)v}-\overset{˜}{g}\left({\overset{˜}{\mu }}^{\left(i\right)}\right)-\Lambda^{\left(i\right)z}\xi^{\left(i\right)v}\\ \xi^{\left(i\right)x} & = & D{\overset{˜}{\mu }}^{\left(i\right)x}-\overset{˜}{f}\left({\overset{˜}{\mu }}^{\left(i\right)}\right)-\Lambda^{\left(i\right)w}\xi^{\left(i\right)x}\text{.} \end{array}$$

**Fig. 1:**
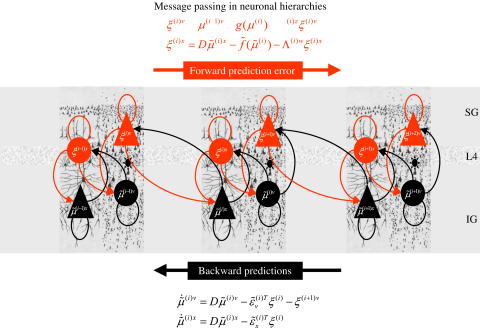
Schematic detailing the neuronal architectures that encode a recognition density on the states of a hierarchical model. This schematic shows the speculative cells of origin of forward driving connections that convey prediction error from a lower area to a higher area and the backward connections that are used to construct predictions. These predictions try to explain away input from lower areas by suppressing prediction error. In this scheme, the sources of forward connections are superficial pyramidal cell populations and the sources of backward connections are deep pyramidal cell populations. The differential equations relate to the optimisation scheme detailed in the main text. The state-units and their efferents are in black and the error-units in red, with causal states on the right and hidden states on the left. For simplicity, we have assumed the output of each level is a function of, and only of, the hidden states. This induces a hierarchy over levels and, within each level, a hierarchical relationship between states, where causal states predict hidden states. This schematic shows how the neuronal populations may be deployed hierarchically within three cortical areas (or macro-columns). Within each area the cells are shown in relation to the laminar structure of the cortex that includes supra-granular (**SG**) granular (**L4**) and infra-granular (**IG**) layers. (For interpretation of the references to colour in this figure legend, the reader is referred to the web version of this article.)

This shows that error-units receive messages from the states in the same level and the level above, whereas states are driven by error-units in the same level and the level below (see Fig. 1). Critically, inference requires only the prediction error from the lower level $\xi^{\left(i\right)}$ and the level in question, $\xi^{\left(i+1\right)}$. These provide bottom-up and lateral messages that drive conditional expectations ${\overset{˜}{\mu }}^{\left(i\right)}$ towards a better prediction, to explain away the prediction error in the level below. These top-down and lateral predictions correspond to ${\overset{˜}{g}}^{\left(i\right)}$ and ${\overset{˜}{f}}^{\left(i\right)}$. This is the essence of recurrent message-passing between hierarchical levels to optimise free-energy or suppress prediction error; i.e., recognition dynamics. In summary, all connections between error- and state-units are reciprocal but the only connections that link levels are forward connections conveying prediction error to state-units and reciprocal backward connections that mediate predictions. This sort of scheme is referred to as predictive coding (Rao & Ballard, 1998).

We can identify error-units with superficial pyramidal cells, because the only messages that pass up the hierarchy are prediction errors and superficial pyramidal cells originate forward connections in the brain. This is useful because it is these cells that are primarily responsible for electroencephalographic (EEG) signals that can be measured non-invasively. Similarly, the only messages that are passed down the hierarchy are the predictions from state-units that are necessary to form prediction errors in lower levels. The sources of extrinsic backward connections are deep pyramidal cells; suggesting that these encode the expected causes of sensory states (see Mumford, 1992 and Fig. 1). Critically, the motion of each state-unit is a linear mixture of bottom-up prediction error (see Eq. (17)). This is exactly what is observed physiologically; bottom-up driving inputs elicit obligatory responses that do not depend on other bottom-up inputs. The prediction error itself is formed by predictions conveyed by backward and lateral connections. These influences embody the nonlinearities implicit in ${\overset{˜}{g}}^{\left(i\right)}$ and ${\overset{˜}{f}}^{\left(i\right)}$. Again, this is entirely consistent with the nonlinear or modulatory characteristics of backward connections.

It has been shown recently that hierarchical architectures (cf, Fig. 1) can be reformulated as a specific type of biased competition, where state-units receive messages from lower-level error-units and direct inputs from higher-level state-units (replacing lateral inputs from error-units in the original predictive coding scheme based on Kalman filtering; Rao & Ballard, 1998). It has been argued that this architecture provides a more realistic model of backward connections in cortex (Spratling, 2008a, 2008b) and usefully connects predictive coding, Kalman filtering and biased competition.

A related Bayesian algorithm called belief-propagation (Dean, 2006; Hinton, Osindero, & Teh, 2006; Lee & Mumford, 2003; Rao, 2006) also rests on message-passing. In these schemes, the messages are not prediction errors but, like prediction errors, are defined self-consistently, in terms of likelihoods and empirical priors. Critically, the belief-propagation algorithm can be derived by minimising free-energy (Yedidia, Freeman, & Weiss, 2005); for example, it can be shown that the Kalman filter is a special case of belief-propagation. This speaks to formal similarities between predictive coding, Bayesian filtering and belief-propagation, which could be implemented by recursive message-passing in the brain and understood in terms of free-energy optimisation.

### 3.3 Summary

In summary, we have seen how the inversion of a generic hierarchical and dynamical model of sensory inputs can be transcribed onto neuronal quantities that optimise a free-energy bound on surprise. This optimisation corresponds, under some simplifying assumptions, to suppression of prediction error at all levels in a cortical hierarchy. This suppression rests upon a balance between bottom-up (prediction error) and top-down (empirical prior) influences. In the final section, we use this scheme to simulate neuronal responses. Specifically, we look at the electrophysiological correlates of prediction error and ask whether we can understand some common phenomena in event-related potential (ERP) research in terms of the free-energy formulation and message-passing in the brain.

## 4 Birdsong and attractors

In this section, we examine a system that uses hierarchical dynamics as a generative model of sensory input. The aim of this section is to provide some face-validity for the functional deconstruction of extrinsic and intrinsic circuits in the previous section. To do this, we try to show how empirical measures of neuronal processes can be reproduced using simulations based on the theoretical analysis above. The example we use is birdsong and the empirical measures we focus on are local field potentials (LFP) or evoked (ERP) responses that can be recorded non-invasively. The material in section is based on the simulations described in Friston and Kiebel (2009).

We first describe our model of birdsong and demonstrate the nature and form of this model through simulated lesion experiments. We then use simplified versions of this model to show how attractors can be used to categorise sequences of stimuli quickly and efficiently. Throughout this section, we will exploit the fact that superficial pyramidal cells are the major contributors to observed LFP and ERP signals. This means we can ascribe these signals to prediction error; because the superficial pyramidal cells are the source of bottom-up messages in the brain (see Fig. 1).

### 4.1 Attractors in the brain

The basic idea here is that the environment unfolds as an ordered sequence of spatiotemporal dynamics (George & Hawkins, 2005; Kiebel, Daunizeau, & Friston, 2008), whose equations of motion entail attractor manifolds that contain sensory trajectories. Critically, the shape of the manifold generating sensory data is itself changed by other dynamical systems that could have their own attractors. If we consider the brain has a generative model of these coupled dynamical systems, then we would expect to see attractors in neuronal dynamics that are trying to predict sensory input. In a hierarchical setting, the states of a high-level attractor enter the equations of motion of a low-level attractor in a nonlinear way, to change the shape of its manifold. This form of generative model has some key characteristics:

First, any level in the model can generate and therefore encode structured sequences of events, as the states flow over different parts of the manifold. These sequences can be simple, such as the quasi-periodic attractors of central pattern generators (McCrea & Rybak, 2008) or can exhibit complicated sequences of the sort associated with chaotic and itinerant dynamics (e.g., Breakspear & Stam, 2005; Canolty et al., 2006; Friston, 1997; Haken, Kelso, Fuchs, & Pandya, 1990; Jirsa, Fuchs, & Kelso, 1998; Kopell, Ermentrout, Whittington, & Traub, 2000; Rabinovich, Huerta, & Laurent, 2008). The notion of attractors as the basis of generative models extends the notion of encoding trajectories in terms of generalised motion, to families of trajectories that lie on the attractor manifold. Hierarchically deployed attractors enable the brain to generate and therefore predict or represent different categories of sequences. This is because any low-level attractor encodes a family of trajectories that correspond to a structured sequence. The neuronal activity representing the trajectory at any one time determines *where* the current dynamics are within the sequence, while the shape of the attractor manifold determines *which* sequence is currently being expressed.

Secondly, if the states in a higher attractor change the manifold of a subordinate attractor, then the states of the higher attractor come to encode the category of the sequence represented by the lower attractor. This means it is possible to generate and represent sequences of sequences and, by induction sequences of sequences of sequences *etc*. This rests upon the states of neuronal attractors at any cortical level providing control parameters for attractors below. This necessarily entails a nonlinear interaction between the top-down effects of the higher attractor and the states of the lower attractor. Again, this is entirely consistent with the nonlinear effects of top-down connections in the real brain.

Finally, this particular model has implications for the temporal structure of perception. Put simply, the dynamics of high-level representations unfold more slowly than the dynamics of lower-level representations. This is because the state of a higher attractor prescribes a manifold that guides the flow of lower states, which could change quite rapidly. We will see an example of this below when considering the perceptual categorisation of different sequences of chirps subtending birdsongs. This suggests that neuronal representations in the brain will change more slowly at higher levels (Kiebel et al., 2008; see also Botvinick, 2007; Hasson, Yang, Vallines, Heeger, & Rubin, 2008). One can turn this argument on its head and use the fact that we are able to recognise sequences of sequences (e.g., Chait, Poeppel, de Cheveigné, & Simon, 2007) as an existence proof for this sort of generative model. In the examples below, we will try to show how autonomous dynamics furnish generative models of sensory input, which behave much like real brains, when measured electrophysiologically.

### 4.2 A synthetic avian brain

**Fig. 2:**
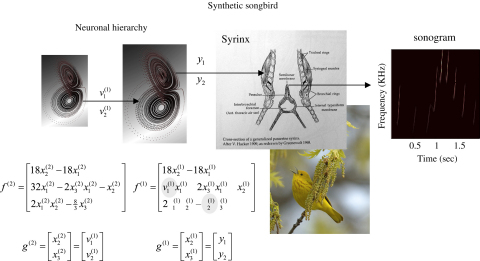
Schematic showing the construction of the generative model for birdsongs. This comprises two Lorenz attractors where the higher attractor delivers two control parameters (grey circles) to a lower-level attractor, which, in turn, delivers two control parameters to a synthetic syrinx to produce amplitude and frequency modulated stimuli. This stimulus is represented as a sonogram in the right panel. The equations represent the hierarchical dynamic model in the form of Eq. (13).

**Fig. 3:**
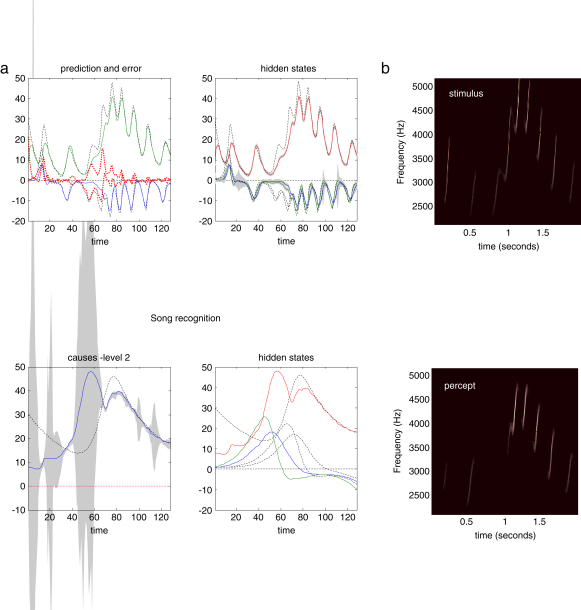
Results of an inversion or deconvolution of the sonogram shown in the previous figure. (a) Upper panels show the time courses of hidden and causal states. Upper left: These are the true and predicted states driving the syrinx and are simple mappings from two of the three hidden states of the first-level attractor. The coloured lines respond to the conditional mean and the dotted lines to the true values. The discrepancy is the prediction error and is shown as a broken red line. Upper right: The true and estimated hidden states of the first-level attractor. Note that the third hidden state has to be inferred from the sensory data. Confidence intervals on the conditional expectations are shown in grey and demonstrate a high degree of confidence, because a low level of sensory noise was used in these simulations. The panels below show the corresponding causal and hidden states at the second level. Again the conditional expectations are shown as coloured lines and the true values as broken lines. Note the inflated conditional confidence interval halfway through the song when the third and fourth chirps are misperceived. (b) The stimulus and percept in sonogram format, detailing the expression of different frequencies generated over peristimulus time. (For interpretation of the references to colour in this figure legend, the reader is referred to the web version of this article.)

The toy example used here deals with the generation and recognition of birdsongs (Laje & Mindlin, 2002). We imagine that birdsongs are produced by two time-varying causal states that control the frequency and amplitude of vibrations of the syrinx of a songbird (see Fig. 2). There has been an extensive modelling effort using attractor models at the biomechanical level to understand the generation of birdsong (e.g., Laje, Gardner, & Mindlin, 2002). Here we use the attractors at a higher level to provide time-varying control over the resulting sonograms. We drive the syrinx with two states of a Lorenz attractor, one controlling the frequency (between two to five kHz) and the other (after rectification) controlling the amplitude or volume. The parameters of the Lorenz attractor were chosen to generate a short sequence of chirps every second or so. To endow the generative model with a hierarchical structure, we placed a second Lorenz attractor, whose dynamics were an order of magnitude slower, over the first. The states of the slower attractor entered as control parameters (the Raleigh and Prandtl number) to control the dynamics exhibited by the first. These dynamics could range from a fixed-point attractor, where the states of the first are all zero; through to quasi-periodic and chaotic behaviour, when the value of the Raleigh number exceeds an appropriate threshold (about twenty four) and induces a bifurcation. Because higher states evolve more slowly, they switch the lower attractor on and off, generating distinct songs, where each song comprises a series of distinct chirps (see Fig. 3).

### 4.3 Song recognition

This model generates spontaneous sequences of songs using autonomous dynamics. We generated a single song, corresponding roughly to a cycle of the higher attractor and then inverted the ensuing sonogram (summarised as peak amplitude and volume) using the message-passing scheme described in the previous section. The results are shown in Fig. 3 and demonstrate that, after several hundred milliseconds, the veridical hidden states and supraordinate causal states can be recovered. Interestingly, the third chirp is not perceived, in that the first-level prediction error was not sufficient to overcome the dynamical and structural priors of the model. However, once the subsequent chirp had been predicted correctly the following sequence of chirps was recognised with a high degree of conditional confidence. Note that when the second and third chirps in the sequence are not recognised, first-level prediction error is high and the conditional confidence about the causal states at the second level is low (reflected in the wide 90% confidence intervals). Heuristically, this means that the synthetic bird listening to the song did not know which song was being emitted and was unable to predict subsequent chirps.

#### 4.3.1 Structural and dynamic priors

**Fig. 4:**
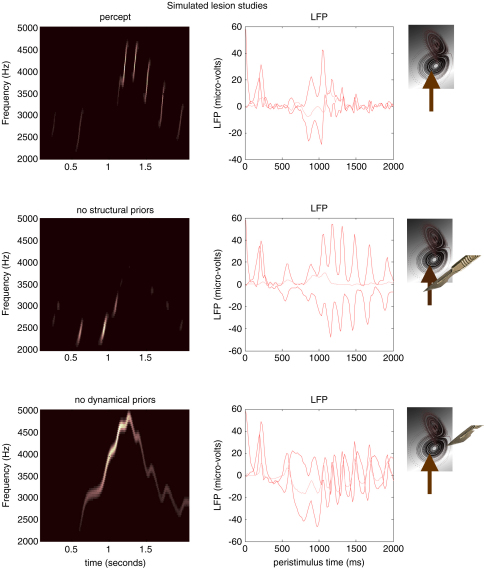
Results of simulated lesion studies using the birdsong model of the previous figure. The left panels show the percept in terms of the predicted sonograms and the right panels show the corresponding prediction error (at the both levels); these are the differences between the incoming sensory information and the prediction and the discrepancy between the conditional expectation of the second level cause and that predicted by the second-level hidden states. Top row: the recognition dynamics in the intact bird. Middle row: the percept and corresponding prediction errors when the connections between the hidden states at the second level and their corresponding causes are removed. This effectively removes structural priors on the evolution of the attractor manifold prescribing the sensory dynamics at the first level. Lower panels: the effects of retaining the structural priors but removing the dynamical priors by cutting the connections that mediate inversion in generalised coordinates. These results suggest that both structural and dynamical priors are necessary for veridical perception.

This example provides a nice opportunity to illustrate the relative roles of structural and dynamic priors. Structural priors are provided by the top-down inputs that reshape the manifold of the low-level attractor. However, this attractor itself contains an abundance of dynamical priors that unfold in generalised coordinates. Both provide important constraints on the evolution of sensory states, which facilitate recognition. We can selectively destroy these priors by lesioning the top-down connections to remove structural priors or by cutting the intrinsic connections that mediate dynamic priors. The latter involves cutting the self-connections in Fig. 1, among the causal and state-units. The results of these two simulated lesion experiments are shown in Fig. 4. The top panel shows the percept as in the previous panel, in terms of the predicted sonogram and prediction error at the first and second level. The subsequent two panels show exactly the same things but without structural (middle) and dynamic (lower) priors. In both cases, the synthetic bird fails to recognise the sequence with a corresponding inflation of prediction error, particularly at the sensory level. Interestingly, the removal of structural priors has a less marked effect on recognition than removing the dynamical priors. Without dynamical priors there is a failure to segment the sensory stream and, although there is a preservation of frequency tracking, the dynamics *per se* have completely lost their tempo. Although it is interesting to compare and contrast the relative roles of structural and dynamics priors; the important message here is that both are necessary for veridical perception and that destruction of either leads to suboptimal inference. Both of these empirical priors prescribe dynamics which enable the synthetic bird to predict what will be heard next. This leads to the question ‘what would happen if the song terminated prematurely?’

### 4.4 Omission-related responses

**Fig. 5:**
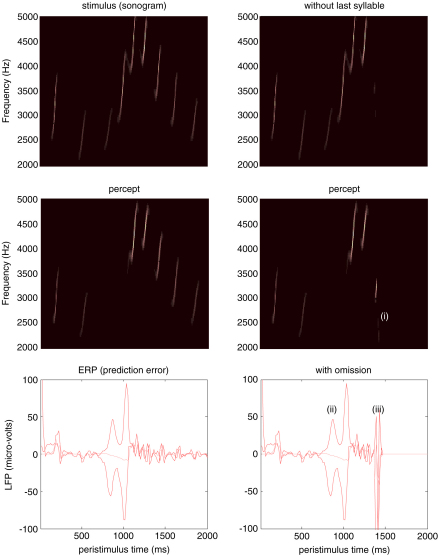
Omission-related responses: Here, we have omitted the last few chirps from the stimulus. The left hand panels show the original sequence and responses evoked. The right hand panels show the equivalent dynamics on omission of the last chirps. The top panels show the stimulus and the middle panels the corresponding percept in sonogram format. The interesting thing to note here is the occurrence of an anomalous percept after termination of the song on the lower right (i). This corresponds roughly to the chirp that would have been perceived in the absence of omission. The lower panels show the corresponding (precision-weighted) prediction error under the two stimuli at both levels. A comparison of the two reveals a burst of prediction error when a stimulus is missed (ii) and at the point that the stimulus terminates (iii) despite the fact that there is no stimulus present at this time. The red lines correspond to prediction error at the first level and the pink lines correspond to prediction error at the second level. (For interpretation of the references to colour in this figure legend, the reader is referred to the web version of this article.)

We repeated the above simulation but terminated the song after the fifth chirp. The corresponding sonograms and percepts are shown with their prediction errors in Fig. 5. The left panels show the stimulus and percept as in Fig. 4, while the right panels show the stimulus and responses to omission of the last syllables. These results illustrate two important phenomena. First, there is a vigorous expression of prediction error after the song terminates prematurely. This reflects the dynamical nature of the recognition process because, at this point, there is no sensory input to predict. In other words, the prediction error is generated entirely by the predictions afforded by the dynamic model of sensory input. It can be seen that this prediction error (with a percept but no stimulus) is almost as large as the prediction error associated with the third and fourth stimuli that are not perceived (stimulus but no percept). Second, it can be seen that there is a transient percept, when the omitted chirp should have occurred. Its frequency is slightly too low but its timing is preserved, in relation to the expected stimulus train. This is an interesting stimulation from the point of view of ERP studies of omission-related responses. These simulations and related empirical studies (e.g., Nordby, Hammerborg, Roth, & Hugdahl, 1994; Yabe, Tervaniemi, Reinikainen, & Näätänen, 1997) provide clear evidence for the predictive capacity of the brain. In this example, prediction rests upon the internal construction of an attractor manifold that defines a family of trajectories, each corresponding to the realisation of a particular song. In the last simulation we look more closely at perceptual categorisation of these songs.

### 4.5 Perceptual categorisation

**Fig. 6:**
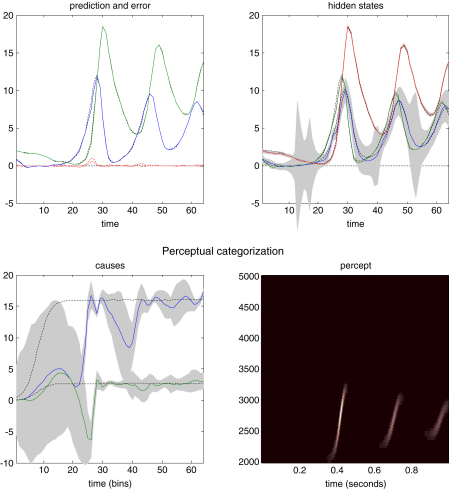
Schematic demonstration of perceptual categorisation. This figure follows the same format as Fig. 3. However, here there are no hidden states at the second level and the causal states were subject to stationary and uninformative priors. This song was generated by a first-level attractor with fixed control variables of $v_{1}^{\left(1\right)}=16$ and $v_{2}^{\left(1\right)}=8/3$ respectively. It can be seen that, on inversion of this model, these two control variables, corresponding to causal states at the second level are recovered with relatively high conditional precision. However, it takes about 50 iterations (about 600 ms) before they stabilise. In other words, the sensory sequence has been mapped correctly to a point in perceptual space after the occurrence of the second chirp. This song corresponds to song C in the next figure.

**Fig. 7:**
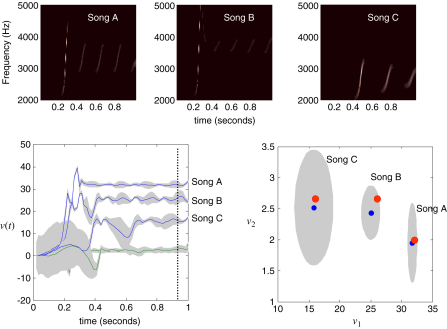
The results of inversion for three songs, each produced with three distinct pairs of values for the second-level causal states (the Raleigh and *Prandtl* variables of the first-level attractor). Upper panel: the three songs shown in sonogram format corresponding to a series of relatively high-frequency chirps that fall progressively in both frequency and number as the Raleigh number is decreased. Lower left: Inferred second-level causal states (blue lines — *Raleigh* and green lines — *Prandtl*) shown as a function of peristimulus time for the three songs. It can be seen that the causal states are identified with high conditional precision after about 600 ms. Lower right: this shows the conditional density on the causal states shortly before the end of peristimulus time (dotted line on the left). The blue dots correspond to conditional means or expectations and the grey areas correspond to the conditional confidence regions. Note that these encompass the true values (red dots) used to generate the songs. These results indicate that there has been a successful categorisation, in the sense that there is no ambiguity (from the point of view of the synthetic bird) about which song was heard.

In the previous simulations, we saw that a song corresponds to a sequence of chirps that are preordained by the shape of an attractor manifold that is controlled by top-down inputs. This means that for every point in the state-space of the higher attractor there is a corresponding manifold or category of song. In other words, recognising or categorising a particular song corresponds to finding a fixed location in the higher state-space. This provides a nice metaphor for perceptual categorisation; because the neuronal states of the higher attractor represent, implicitly, a category of song. Inverting the generative model means that, probabilistically, we can map from a sequence of sensory events to a point in some perceptual space; where this mapping corresponds to perceptual recognition or categorisation. This can be demonstrated in our synthetic songbird by ignoring the dynamics of the second-level attractor and exposing the bird to a song and letting the states at the second level optimise their location in perceptual space. To illustrate this, we generated three songs by fixing the Raleigh and Prandtl variables to three distinct values. We then placed uninformative priors on the second-level causal states (that were previously driven by the hidden states of the second-level attractor) and inverted the model in the usual way. Fig. 6 shows the results of this simulation for a single song. This song comprises a series of relatively low-frequency chirps emitted every 250 ms or so. The causal states of this song (song C in the next figure) are recovered after the second chirp, with relatively tight confidence intervals (the blue and green lines in the lower left panel). We then repeated this exercise for three songs. The results are shown in Fig. 7. The songs are portrayed in sonogram format in the top panels and the inferred perceptual causal states in the bottom panels. The left panel shows the evolution of the causal states for all three songs as a function of peristimulus time and the right panel shows the corresponding conditional density in the causal or perceptual space of these two states after convergence. It can be seen that for all three songs, the 90% confidence interval encompasses the true values (red dots). Furthermore, there is very little overlap between the conditional densities (grey regions), which means that the precision of the perceptual categorisation is almost 100%. This is a simple but nice example of perceptual categorisation, where sequences of sensory events with extended temporal support can be mapped to locations in perceptual space, through Bayesian deconvolution of the sort entailed by the free-energy formulation.

## 5 Conclusion

This paper has suggested that the architecture of cortical circuits speaks to hierarchical generative models in the brain. The estimation or inversion of these models corresponds to a generalised deconvolution of sensory inputs to disclose their causes. This deconvolution could be implemented in a neuronally plausible fashion, where neuronal dynamics self-organise when exposed to inputs to suppress free-energy. The focus of this paper has been on the nature of the hierarchical models and, in particular, how one can understand message-passing among neuronal populations in terms of perception. We have tried to demonstrate their plausibility, in relation to empirical observations, by interpreting the prediction error, associated with model inversion, with observed electrophysiological responses.

The ideas reviewed in this paper have a long history, starting with the notion of neuronal energy (Helmholtz, 1860/1962); covering ideas like efficient coding and analysis by synthesis (Barlow, 1961; Neisser, 1967) to more recent formulations in terms of Bayesian inversion and Predictive coding (e.g., Ballard, Hinton, & Sejnowski, 1983; Dayan, Hinton, & Neal, 1995; Kawato, Hayakawa, & Inui, 1993; Mumford, 1992; Rao & Ballard, 1998). This work has also tried to provide support for the notion that the brain uses dynamics to represent and predict causes in the sensorium (Byrne, Becker, & Burgess, 2007; Deco & Rolls, 2003; Freeman, 1987; Tsodyks, 1999).
